# Detachable Robotic Grippers for Human-Robot Collaboration

**DOI:** 10.3389/frobt.2021.644532

**Published:** 2021-06-17

**Authors:** Zubair Iqbal, Maria Pozzi, Domenico Prattichizzo, Gionata Salvietti

**Affiliations:** ^1^ Department of Information Engineering and Mathematics, University of Siena, Siena, Italy; ^2^ Istituto Italiano di Tecnologia, Genoa, Italy

**Keywords:** human-robot collaboration, collaborative grippers, soft grippers, tool changers, wearable interfaces

## Abstract

Collaborative robots promise to add flexibility to production cells thanks to the fact that they can work not only *close to* humans but also *with* humans. The possibility of a direct physical interaction between humans and robots allows to perform operations that were inconceivable with industrial robots. Collaborative soft grippers have been recently introduced to extend this possibility beyond the robot end-effector, making humans able to directly act on robotic hands. In this work, we propose to exploit collaborative grippers in a novel paradigm in which these devices can be easily attached and detached from the robot arm and used also independently from it. This is possible only with self-powered hands, that are still quite uncommon in the market. In the presented paradigm not only hands can be attached/detached to/from the robot end-effector as if they were simple tools, but they can also remain active and fully functional after detachment. This ensures all the advantages brought in by tool changers, that allow for quick and possibly automatic tool exchange at the robot end-effector, but also gives the possibility of using the hand capabilities and degrees of freedom without the need of an arm or of external power supplies. In this paper, the concept of *detachable robotic grippers* is introduced and demonstrated through two illustrative tasks conducted with a new tool changer designed for collaborative grippers. The novel tool changer embeds electromagnets that are used to add safety during attach/detach operations. The activation of the electromagnets is controlled through a wearable interface capable of providing tactile feedback. The usability of the system is confirmed by the evaluations of 12 users.

## 1 Introduction

The introduction of collaborative robot manipulators, capable of safely sharing the workspace with humans, has represented a step change in robotics and paved the way to a variety of new human-robot collaboration (HRC) paradigms (Ajoudani et al., 2018). These go beyond the mere suppression of cages and replacement of industrial robots with collaborative ones, as the fact that humans and robots can come into contact (voluntarily or involuntarily) not only requires additional considerations in terms of safety, but also enables previously inconceivable applications (Kumar et al., 2021).

A safe coexistence of humans and robots is mainly obtained by implementing collision avoidance and contact handling strategies which ensure that the robot avoids the contact with the human as long as possible and behaves safely in case it occurs (De Luca and Flacco, 2012; Haddadin and Croft, 2016). Once safety is ensured, the actual collaboration, intended as a coordination of actions and intentions between humans and robots, can be implemented. The collaboration can be either *physical* or *contactless* (De Luca and Flacco, 2012), and usually requires a certain level of mutual understanding between the human and the robot. On the one hand, the robot needs to be aware of human actions. This can be achieved with methods for predicting human activity patterns (Zanchettin et al., 2018), or for recognizing human intention (Singh et al., 2020; Buerkle et al., 2021). On the other hand, the operator must be informed about the robot activity and the task state. This can be achieved, for example, through the use of suitable interfaces for alerting the user in critical phases of the task (Katayama et al., 2020).

Many works in the field of HRC have focused on the control of the robot arm, but it is also important to study how to properly design and control the end-effector that is attached to it. Research works on soft robotic hands go in this direction (Piazza et al., 2019) and also devices which are expressly thought for HRC have been proposed. An example is the SofTHand Industry (qbrobotics) which complies to standards and certifications of industrial and collaborative robotics^1^. Another recent prototype of collaborative gripper has been proposed by Salvietti et al. (2018) and it is called Co-Gripper. It is modular, reconfigurable, and remotely commanded through a wearable interface. Differently from most of the available robotic hands, the Co-Gripper can also be used detached from the robot manipulator, allowing for easy tool exchange, parallelization of tasks, and avoidance of complex object re-grasping procedures. The device is a fully functional gripper with an on-board battery that guarantees portability and allows to use it even when detached from the robot arm and placed on a passive support Salvietti et al. (2020).

**FIGURE 1:**
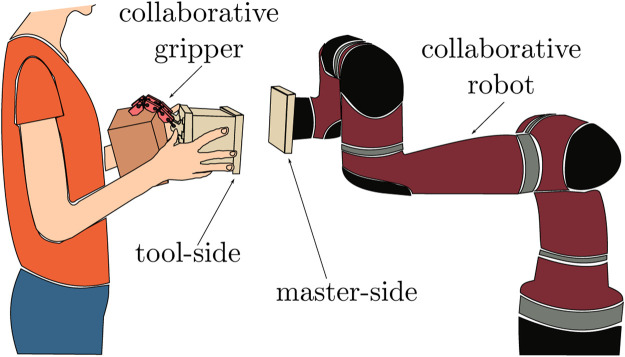
Detachable grippers concept: the user can detach and attach different end-effectors to a collaborative robot arm. The gripper can be detached from the arm while it is holding an object and the user can work on it (*e.g.*, assemble some parts), possibly helped by the robotic hand itself. In the meantime, the robot arm can perform other operations. This scenario is possible only using collaborative self-powered grippers that can be safely handled by humans and do not require external power sources to work.

In this paper, we propose a new paradigm of human-robot collaboration in which modular, detachable, and self-powered robotic grippers are used in combination with customized tool changers to add flexibility to the collaborative task. A sketch of the envisioned scenario is shown in Figure 1.

Tool changers, or quick change end-effectors, are devices which allow to efficiently perform the replacement of tools at the end-effector of a robot manipulator. Whether they are research prototypes or commercial products (*e.g.*, devices by SCHUNK or ATI Industrial Automation), tool changers are usually composed of a Master-side to be attached to the robot and a Tool-side to be attached to the tool. Tool change can be either manual or automatic. The former case requires human intervention to attach and detach tools (McCormick and Beall, 2000), the latter allows robots to directly couple with different tools placed on ad-hoc racks (Silvers, 1986; Meghdari and Barazandeh, 2000). In (Chen et al., 2014), a passive bit-changing mechanism was proposed to add flexibility in a collaborative scenario.

To demonstrate the utility and usability of *detachable robotic grippers*, we designed a new tool changer allowing for safe attach/detach operations. It embeds electromagnets that can be activated and deactivated by the user through push buttons mounted in a wearable interface. The electromagnets allow to quickly connect the gripper tool-side to the master-side, *see* Figure 1. This gives time to the operator to safely lock/unlock the gripper to the robot by means of mechanical draw latch locks. The wearable interface is a ring-shaped device that is also endowed with a vibration motor to give tactile feedback to the human (Salvietti et al., 2020). The wearable human-machine interface is fundamental in the proposed paradigm, as it allows the human to have full control not only on the closing/opening of the robotic gripper, but also on attach/detach operations. The same idea could be applied in highly flexible manufacturing processes exploiting commercially available tool-changers and collaborative grippers.

The rest of the paper is organized as follows. Section 2 introduces the concept and the advantages of having detachable robotic grippers through an illustrative example. Section 3 describes a possible implementation of a tool changer tailored for collaborative grippers and Section 4 presents a user study aimed at evaluating the usability of the proposed system. Section 5 draws the conclusions of the paper and outlines possible future developments of the proposed framework.

## 2 Detachable Grippers Concept

The concept of detachable robotic grippers can introduce several advantages in human-robot collaborative tasks. In fact, a combination of self-powered grippers with suitably designed tool changers allows to: *1*) easily free the robot end-effector, *2*) stably work on the product without the need of an ad-hoc assembly station with specific fixtures, *3*) operate over fragile or delicate objects, *4*) save the time required for objects re-grasping, and *5*) better exploit the possible in-hand dexterity of the gripper.

In the flexible cells envisaged for human-robot collaborative tasks, human and robot arm may have to operate over the same object. The robot arm could, for instance, be used to move a product from a station to another where human workers have to operate on it. Upon completion of human operation, the robot could be employed again to transport the final product to another station. Normally, to allow human mates to work on the object, the robot should either wait for the completion of the work while holding it, or leave it on a safe spot. In the second case, later on, the robot should re-grasp the final product to bring it where needed. The use of detachable hands, instead, would allow human workers to safely remove the gripper and the object held by it, and thus free the robot arm resource. The robot arm can then be used to connect another gripper or a different tool, and perform other operations while waiting for the human to finish. In most of the collaborative tasks implemented at the moment, the human operator rarely hands over objects directly to the robot and thus flexible cells are usually endowed with ad-hoc assembly stations where specific fixtures are designed to simplify grasping operations. Detachable hands would allow to directly use the gripper as a working station, removing the need for fixtures and re-grasp operations by the robot, since the object would always be held by the gripper. This feature may be particularly useful to do some operations with the object even in case it cannot be touched by the human or repositioned with respect to the hand (*e.g.*, very fragile vials that must be filled with liquid and/or transported by the human somewhere, or sterile items that cannot be touched by operators). In the list of advantages at the beginning of this section, we also mentioned the possibility to exploit in-hand dexterity of the gripper during human operations. We imagine that the gripper could perform some in-hand reconfiguration of the object that my be useful for the human work. A simple example could be a gripper able to in-hand rotate an object during the human operation.

**FIGURE 2:**
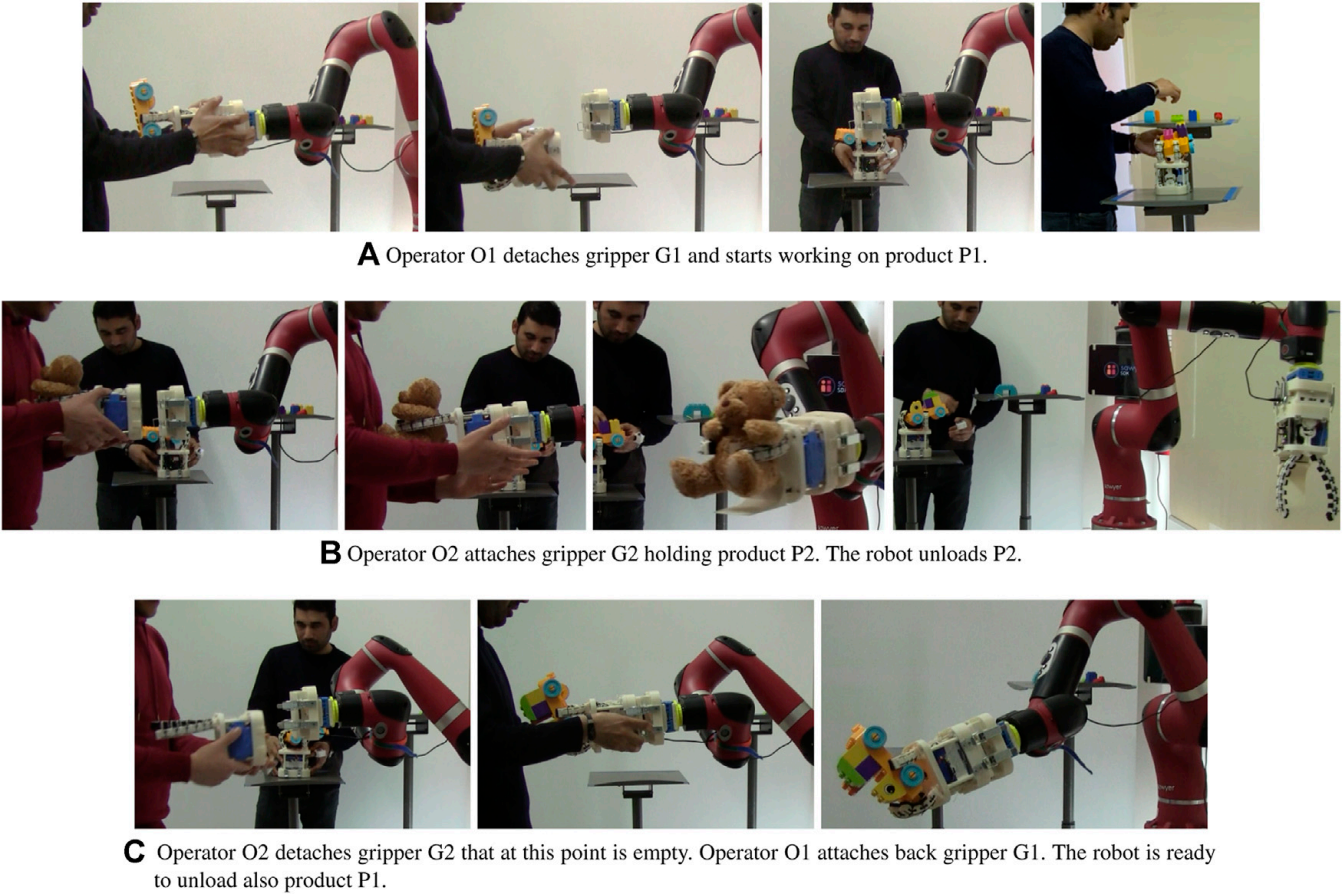
Example of human-robot collaboration task in which some of the advantages of using detachable robotic grippers in collaborative scenarios are shown.

In the following, we will use an illustrative example to describe the possibility of using detachable hands as a mean to improve human-robot collaboration. The full video is available at link^2^. Let us consider a human-robot collaboration scenario in which two different products (P1 and P2) have to be assembled and loaded in a delivery box. Two different operators (O1 and O2) have to work on the products, that have to be picked up with two different collaborative grippers (G1 and G2) and moved by a collaborative robot arm. The arm is endowed with the master-side of a quick change end-effector that can be attached to different grippers and tools. This fictional scenario is reproduced in the video sequences displayed in Figure 2. In Figure 2A, the robot brings a part of product P1 held by gripper G1 to operator O1, that detaches G1 and starts working to assemble P1, doing operations that might be difficult for a robot (*e.g.*, handling and assembling highly deformable parts). The next sequence of actions (Figure 2B) shows another operator, O2, that, after having assembled product P2, attaches gripper G2 holding P2 to the collaborative robot. After that, the robot unloads the product and goes back towards operator O2. In the meantime, O2 is free to do other tasks and O1 continues working on P1. In the last part of the task (Figure 2C), operator O2 detaches G2 and operator O1 attaches back gripper G1 to the robot. At this point, the robot is ready to unload also product P1. Note that at the end of the first sequence of actions (Figure 2A) the gripper remains attached to the object while the human operates over it. In this situation, all or part of the advantages listed at the beginning of this section can be exploited.

In the presented example, an assembly task is performed and the setup is composed of two grippers, the Co-Gripper (Salvietti et al., 2018) and the Soft ScoopGripper (Salvietti et al., 2019), a collaborative robot arm, the Sawyer Robot (Rethink Robotics), and a customized tool changer, that is described in Section 3. The scenario shown in Figure 2, however, is just used to give an idea of the possibilities offered by detachable grippers in highly flexible human-robot collaborative tasks, and can be generalized to other applications and other devices.

**FIGURE 3:**
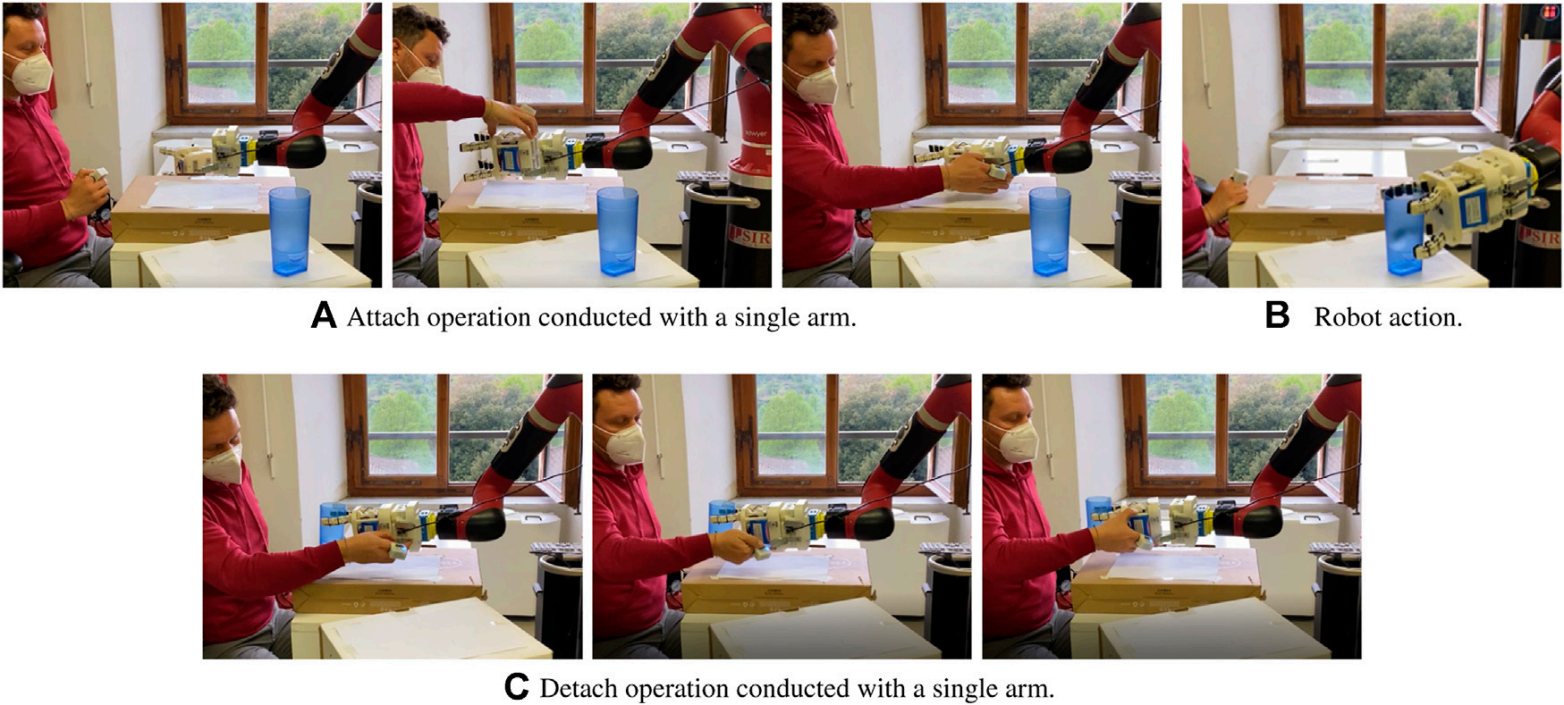
Use of a detachable robotic gripper in an assistive scenario in which the user has lost the mobility of an arm. All the attach/detach operation can be executed with the help of the magnetic connection using only one arm.

Collaborative robots can also be used in assistive applications to help people with reduced mobility (Vogel et al., 2020). In this context, having a platform that allows the easy exchange of tools at the robot end-effector can be useful for people who lost an arm (e.g., amputees) or have reduced mobility in it (e.g., post-stroke patients), and thus should be able to attach/detach tools with a single hand. Figure 3 shows an example in which attach and detach operations are performed with one hand. In this case, the adopted tool changer should be properly designed and the tool changer design described in Section 3 is particularly suitable, as the embedded magnets are fundamental to accomplish attach/detach operations.

## 3 Tool Changer for a Collaborative Gripper

In this section, we propose a possible implementation of a tool changer for collaborative detachable grippers. The master side of the device is endowed with electromagnets and draw latches. Electromagnets are exploited during attach/detach phases to guide the user in the alignment of tool and master sides and to support the weight of the gripper while the operator uses a mechanical locking system (four draw latches in our case) to fix the gripper to the robot. Once the gripper is firmly attached through the latches, it is ready to perform grasping and manipulation tasks, and electromagnets can be switched off to avoid power consumption.

Most of the available tool changers use pneumatic systems to attach master and tool sides (Ryuh et al., 2006). While compressed air supply is common in manufacturing and industrial environments, cooperative robots are expected to soon enter domestic and unstructured working places, where compressed air supply might not be available or might introduce constraints to the robot operation. This is why we decided to rely on a different type of technology. There are very few examples of tool changers that exploit electromagnets, and they usually embed also other locking mechanisms (Jones, 1986). The adoption of electromagnets requires minimal changes on the tool side and provides the system with features that are particularly suitable for HRC contexts. First, electromagnets can easily be activated/deactivated depending on the user’s needs. Second, the magnetic force creates a sort of funnel which helps users to well align master and tool sides and increases the safety of the system, that in the most crucial phases can rely on two different locking mechanisms. In addition, while magnets represent an additional safety feature in industrial scenarios, they are fundamental in assistive scenarios where, for example, the user might have mobility problems in an arm and thus should rely entirely on the magnets during attach/detach operations (*see* Figure 3).

### 3.1 Device Design

**FIGURE 4:**
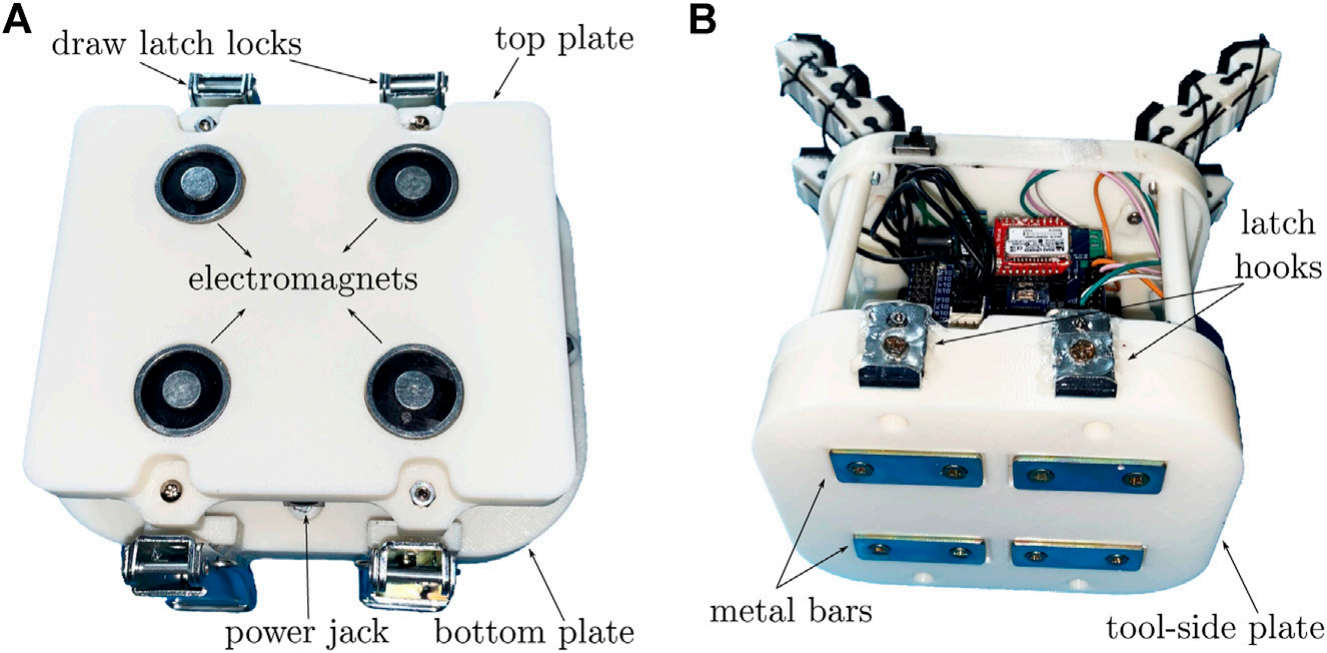
**(A)** Magnetic platform that constitutes the master-side of the presented tool changer. **(B)** Light version of the Co-Gripper (Salvietti et al., 2018) with tool-side plate embedding metal bars and latch hooks.

The two sides of the proposed tool changer are shown in Figure 4. The master-side consists of four electromagnets kits embedded in a 3D-printed plastic cover (*see* Figure 4A). Each electromagnet can absorb a 5 kg ferromagnetic and produces a 50 N suction force. All the electromagnets are controlled by Pulse Width Modulation (PWM) signals coming from a powerful Microcontroller Teensy 3.2, which incorporates MK20DX256 32 bit ARM Cortex-M4 72 MHz processing unit.

In our setup, we decided to adopt the Co-Gripper that is well suited to work with a tool changer because it can be commanded wireless and is self-powered. It is composed of four modular soft fingers that can be reconfigured to obtain different grasps (Salvietti et al., 2018). It embeds two motors, each of which moves a pair of fingers thanks to tendons connected to a differential mechanism. To adapt the Co-Gripper to work with the magnetic platform, metal bars and latch hooks were added to the base of the device, constituting the tool side of our tool changer system (*see* Figure 4B). The gripper weighs 400 g (we used a lighter renovated version with respect to the one presented by Salvietti et al. (2018)), and the magnetic platform can hold a cumulative weight (gripper and object) of 3 kg.

The Co-Gripper has an on-board 12 V, 2600 mAH battery which can withstand 2–3 h of continuous operation. An important feature that can be added to the master-side system is a connection such that, once Co-Gripper is attached to the magnetic platform, it is powered directly from the power coming from the robot or another power source. This can be achieved, for example, by connecting the metal bars to the main power inlet as soon as the user connects the Co-Gripper to the robot arm. In this way, the gripper could work using external power and, possibly, in the meantime, the battery could also be recharged.

### 3.2 Device Control

**FIGURE 5:**
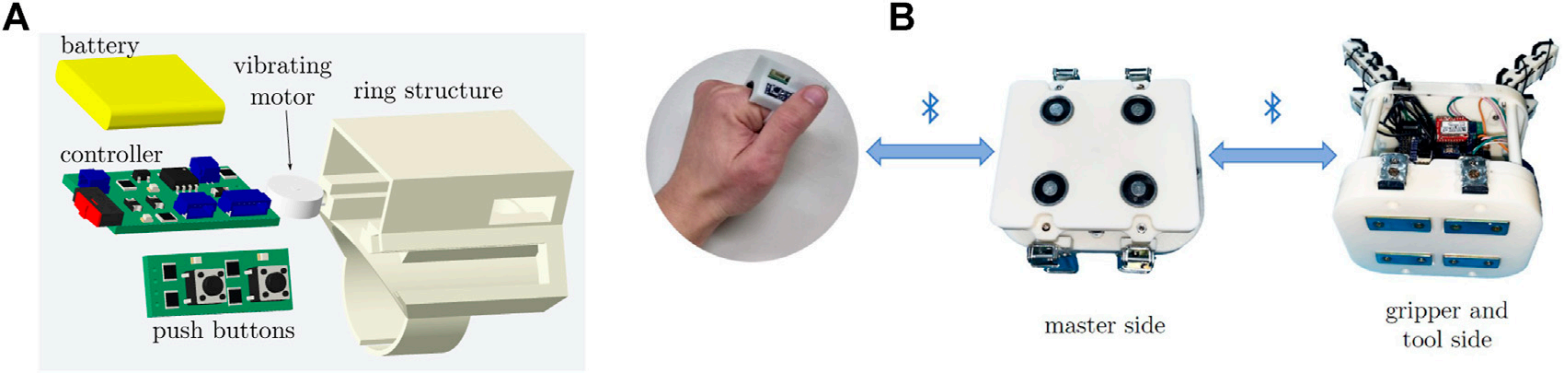
Wearable ring interface. **(A)** Exploded view of its components (Salvietti et al., 2020). Push buttons can be used for controlling the activation of the electromagnets of the tool changer and the opening of the gripper. The ring also embeds a vibrating motor that starts vibrating when the magnets are active. **(B)** Interconnections between the interface and the detachable gripper system. The signals coming from the ring are sent *via* Bluetooth to the control board of the master side of the tool changer which, depending on the pressed button, either switches on/off the magnets, or sends the opening/closing signal to the gripper *via* Bluetooth.

To control the activation of the electromagnets a wireless ring is used (Salvietti et al., 2020) (*see* Figure 5A). The ring contains two push buttons and on board circuitry. A coin type shaft-less vibratory motor (Precision drive, United States) with a diameter of 10 mm is also installed in the ring. RN42-i/rm Bluetooth modules are used to establish the communication between magnetic platform and ring. Two Bluetooth modules are used in the magnetic platform, where one serves the purpose of communicating with the wireless ring and the other one allows the user to control the gripper opening and closing by using the same ring interface (Figure 5B).

The buttons embedded in the ring work as follows. The distal button activates/deactivates all electromagnets. One press of the button activates them, and the next one deactivates them. After activation, a continuous vibrotactile feedback is provided to the user to inform him/her that all the electromagnets are switched on. The proximal button of the ring can be used to open/close the used gripper.

We chose this configuration of buttons and control actions for the sake of simplicity. Other more sophisticated strategies (e.g., multiple pressing of the button, use of proximity sensors for automatic closing, etc…) may be implemented using the same system to avoid accidental activations or to speed-up grasping operations.

## 4 Experimental Results

To investigate whether the proposed device is judged usable by naive users, we carried out a study involving 12 volunteers (age range 25–32, three females, nine males). They gave their informed consent to participate in the experiment and they did not receive any payment and were able to leave the experiment at any moment. After an initial attach/detach operation performed to get acquainted with the system, participants were asked to attach and detach the Co-Gripper six consecutive times using the wearable ring to command the magnets. We asked to go as fast as possible during the single attach or detach operation, whereas participants could wait as long as they wanted between the two. In three out of six trials users got a continuous vibrotactile feedback while the magnets were switched on, the other three trials were conducted without feedback. Half of the participants tested first the “with feedback” (F) condition, whereas the other half tested the “without feedback” (N) condition.

Participants were asked to wear headphones with white noise during the experiments, so to avoid possible influence coming from the low noise produced by the electromagnets when activated.

**FIGURE 6:**
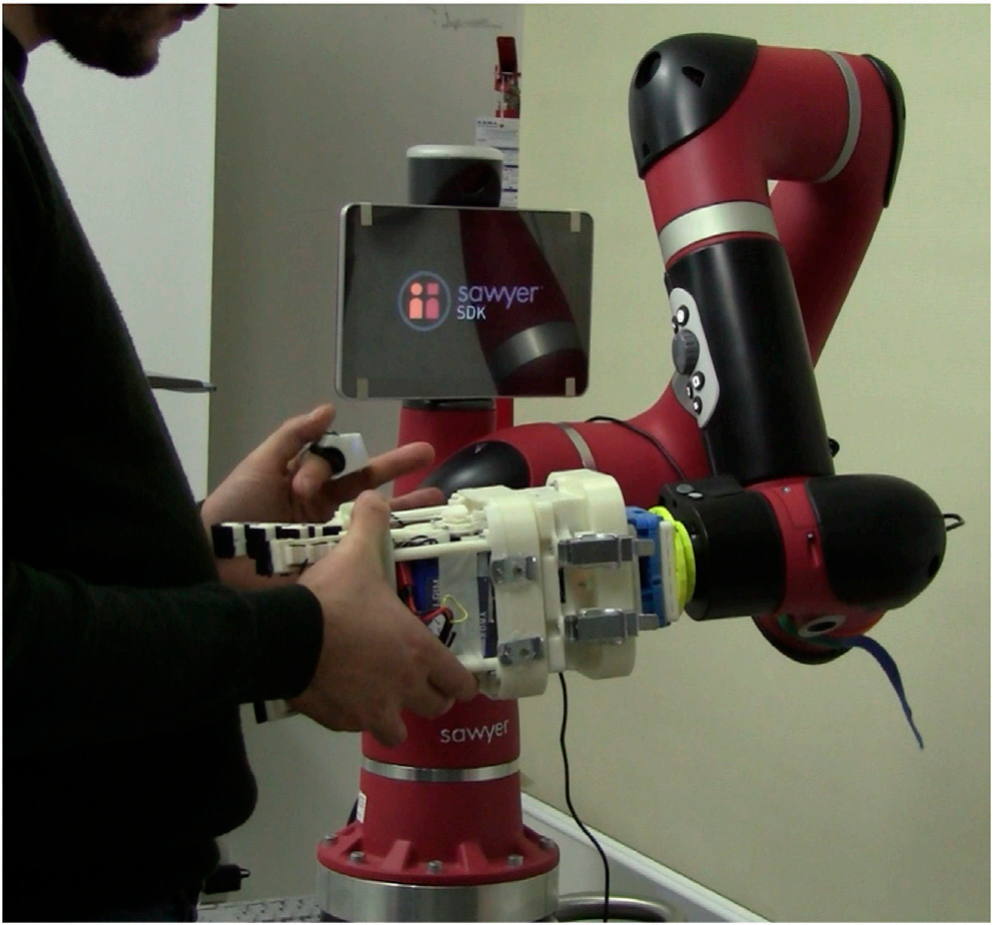
Experimental setup for the user study: the human operator wears the tactile ring and uses the magnetic tool changer to attach/detach the Co-Gripper to/from a collaborative robot arm (Sawyer Robot, Rethink Robotics).

**FIGURE 7:**
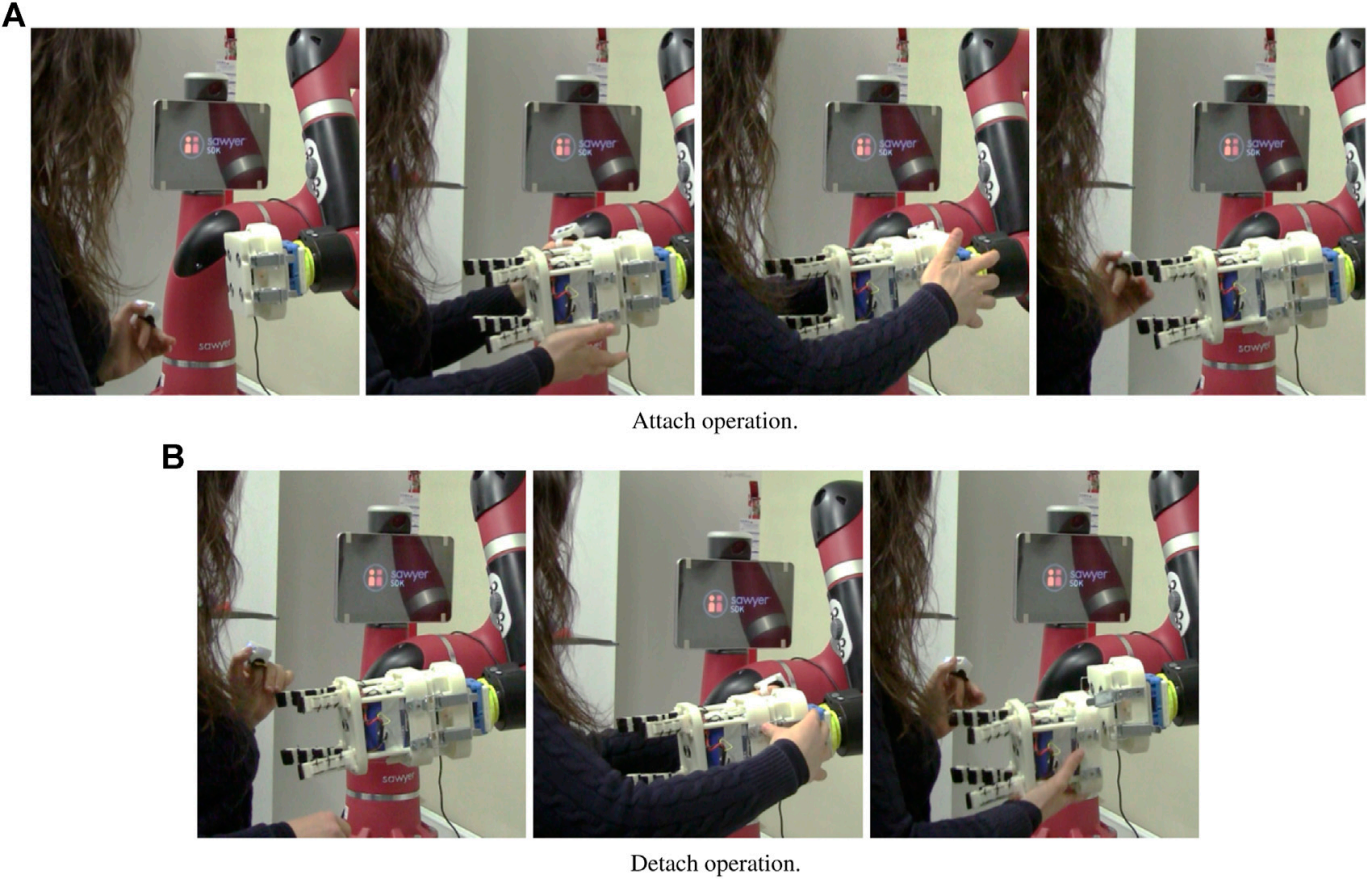
Attach and detach sequences performed by the participants of the usability study.

In Figure 6 and Figure 7, the experimental setup and the sequences of actions needed to attach/detach the gripper are shown, respectively. Note that in the experiments the robot arm was kept fixed and only the tool changer system was used by the participants.

Attaching the gripper to the robot requires to *1*) activate the magnets, *2*) attach the gripper to the magnets, *3*) attach the draw latches, and *4*) deactivate the magnets (*see* Figure 7A). For detaching the gripper, instead, the following actions are needed: *1*) activate the magnets, *2*) detach the draw latches, and *3*) deactivate the magnets while detaching the gripper. Note that when the gripper is magnetically attached, there is a sort of “magnetic buffer” during which user hands are left free to close/open the draw latches. Besides, in the detaching phase, the user can deactivate the magnets whenever he/she feels more comfortable to grasp the gripper.

**FIGURE 8:**
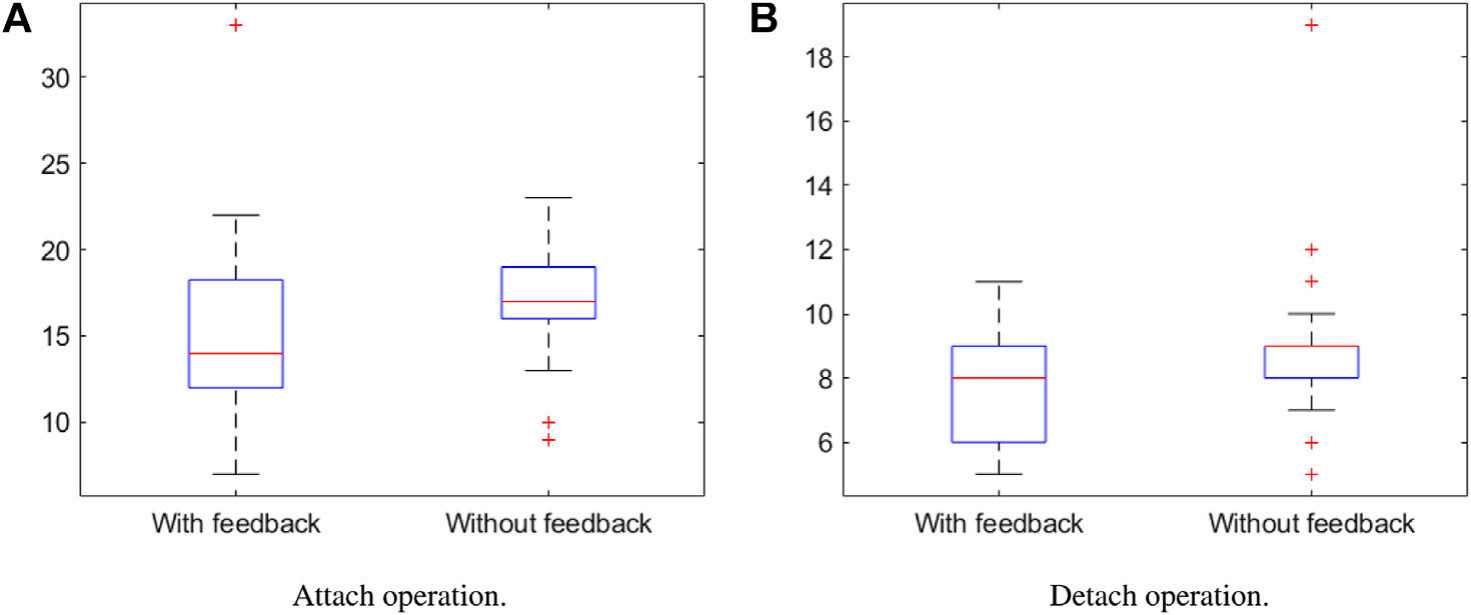
Comparison of completion times with and without the use of tactile feedback. Boxplots of the completion time measured in the experimental trials for **(A)** attach operations and **(B)** detach operations.

During experiments we measured the time for completing attach/detach operations with and without feedback. The obtained results are shown in Figure 8. We found statistical evidence of the fact that the completion time is reduced with the help of vibrotactile feedback. A single-tailed Wilcoxon signed rank test with confidence α=0.05 returns $p=5.0127\text{*}10^{-4},z=-3.2898$ for the attach phase and confidence α=0.05 returns $p=6.6522\text{*}10^{-4},z=-3.2898$ for the detach phase. In general, the attach phase requires more time (mean with feedback = 15.1282 s, without feedback = 17.6923 s) than the detach phase (mean with feedback = 7.5128 s, without feedback = 9.1026 s) as it takes longer to attach the draw latches than to detach them.

**FIGURE 9:**
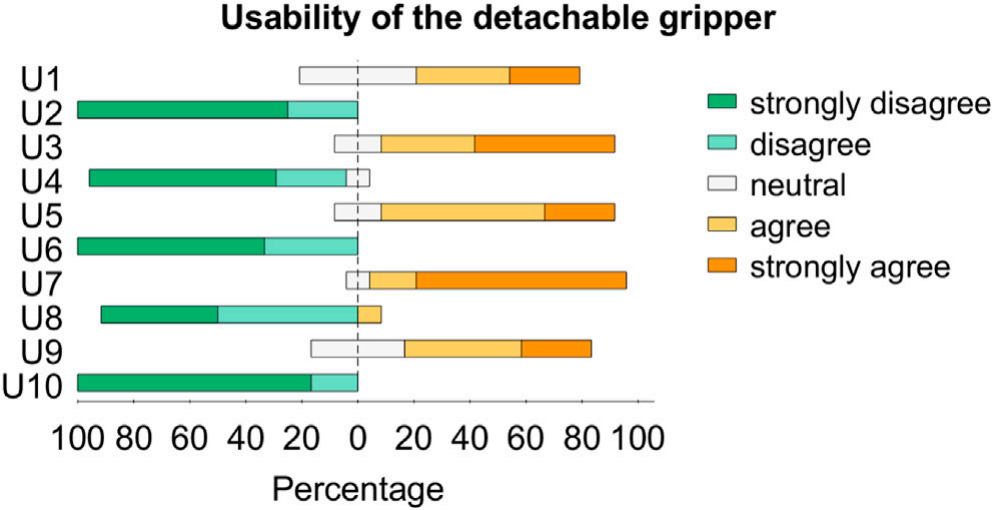
Answers of the 12 users to the questions on usability reported in Table 1.

**FIGURE 10:**
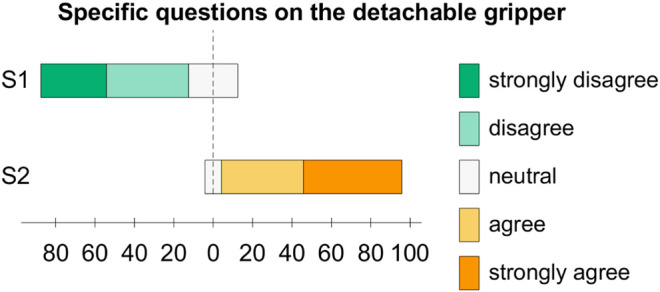
Answers of the 12 users to the specific questions on the system reported in Table 2.

After the completion of the experimental trial, each subject was asked to reply to 12 questions formulated as five-level Likert items, with possible answers varying from “strongly disagree (SD)” to “strongly agree (SA).” The first 10 statements corresponded to the System Usability Scale (SUS) (Brooke, 1996) (U1-U10, Table 1), whereas the last two were more focused on the proposed system features (S1, S2, Table 2). Figure 9 and Figure 10 give an overview of the users’ answers.

**TABLE 1:** List of statements on usability proposed to the users after they tried the system.

	
U1:	I think that I would like to use this system frequently.
U2:	I found this system unnecessarily complex.
U3:	I thought this system was easy to use.
U4:	I think that I would need assistance to be able to use this system.
U5:	I found the various functions in this system were well integrated.
U6:	I thought there was too much inconsistency in this system.
U7:	I would imagine that most people would learn to use this system very quickly.
U8:	I found this system very cumbersome/awkward to use.
U9:	I felt very confident using this system.
U10:	I needed to learn a lot of things before I could get going with this system.

**TABLE 2:** List of specific statements proposed to the users after they tried the system.

	
S1:	I frequently had the impression that the gripper was about to fall while attaching/detaching it.
S2:	I found the vibrotactile feedback provided by the ring useful.

The attach/detach mechanism was designed to be commanded through a wearable interface which also provides vibrotactile feedback related to the status of the platform. This is why participants were asked to answer U1-U10 and S1 thinking about the experiments they performed *with* the feedback, while they had to answer S2 comparing their experience in the F condition with that in the N condition. In other words, we let users try the situation in which the feedback is off to let them realize the usefulness (or not) of the feedback itself.

## 4.1 Discussion

With the conducted experimental trials we found that haptic feedback allows to use the magnetic tool changer in a more efficient way.

Considering the SUS items in Table 1, the system for attaching/detaching the hand got an average score close to 85/100. Since the average SUS score for a usable system is considered to be 68 (Sauro, 2011), our result is well above the average. Separating the questions on Learnability (items 4 and 10) from those on Usability (remaining eight items) as suggested in (Lewis and Sauro, 2009), we get a score close to 93/100 for Learnability and to 83/100 for Usability. One of the participants agreed with U8, but, looking at the other given replies (U1: SA, U2: D, U3: SA, U4: SD, U5: A, U6: SD, U7: SA, U9: SA, U10: SD, S1: SD, S2: SA), this did not influence the overall perceived usability of the system.

Regarding the specific questions on the system for detaching and attaching the gripper, nine out of 12 users disagreed (four strongly) with S1 and the other were neutral, whereas 11 out of 12 agreed with S2 (six strongly) and only one was neutral. Results obtained for S1 show that, overall, people were not concerned about loosing the gripper. Results for S2 clearly demonstrate the importance of having a feedback about the status of the magnets. We chose to provide a haptic feedback as the Co-Gripper system already includes a vibrotactile ring, and haptic cues can be useful when other senses are busy or impaired (Casalino et al., 2018). For instance, in industrial environments, the auditory channel may be impaired by personal protective equipment or by the production noise.

## 5 Conclusions

This paper presents the concept of detachable grippers, a novel human-robot collaboration paradigm in which a further degree of flexibility is introduced by the possibility of quickly locking and unlocking the gripper from the robotic arm. Devices to easily attach and detach tools from industrial robots (tool changers) are widely spread, but here we propose to attach/detach portable grippers that are designed for human-robot collaboration and that can be used even when detached from the robot arm, without external power supply. After the explanation of an illustrative use case, that underlines the possible applications of such collaboration paradigm, a magnetic tool changer tailored for collaborative grippers is described. A user study involving 12 subjects has demonstrated the usability of the proposed system and the importance of tactile feedback both for a more effective use of the magnetic tool changer.

In future work, we intend to replace the manual latch locking with automatic mechanical locking to further decrease the time for attaching and detaching the master and tool sides. We also plan to study more in detail the role of haptic feedback during handover operations in collaborative scenarios.

We envision that our framework could be useful in unstructured scenarios where no ad-hoc assembly stations are present, and where power and compressed air supply are not always or everywhere available. Think, for example, to domestic environments in which the robot could provide assistance in house chores, or small workshops, where robots could help artisans in assembling products. Having self powered grippers coupled with tool changers that can be controlled through an intuitive wearable interface could allow users to adapt and customize the collaborative system according to their needs.

## Data Availability

The original contributions presented in the study are included in the article/Supplementary Material, further inquiries can be directed to the corresponding author.

## Footnotes

1 QB SoftHand Industry: https://qbrobotics.com/it/prodotti/qb-softhand-industry/, accessed: 05-2021.

2 Detachable Gripper video: https://youtu.be/Btaxku537jk.
